# Supplementary material for: Prolyl isomerization controls activation kinetics of a cyclic nucleotide-gated ion channel
Source: Nat Commun. 2020 Dec 16;11:6401. doi: 10.1038/s41467-020-20104-4 (PMC7744796; doi:10.1038/s41467-020-20104-4)
Supplement: Supplementary file 1 — Supplementary Information [file 41467_2020_20104_MOESM1_ESM.pdf]

Prolyl Isomerization Controls Activation Kinetics of a Cyclic Nucleotide-Gated Ion Channel

Supplementary information

Philipp A.M. Schmidpeter[1], Jan Rheinberger[1,2] and Crina M. Nimigean[1,3 *]

[1] Weill Cornell Medicine, Department of Anesthesiology, 1300 York Avenue, New York, NY 10065, USA

[2] present address: University of Groningen, Netherlands

[3] Weill Cornell Medicine, Department of Physiology and Biophysics, 1300 York Avenue, New York, NY 10065, USA

[*] correspondence to: crn2002@med.cornell.edu

Supplementary Figures 1 – 9

Supplementary Tables 1 – 5

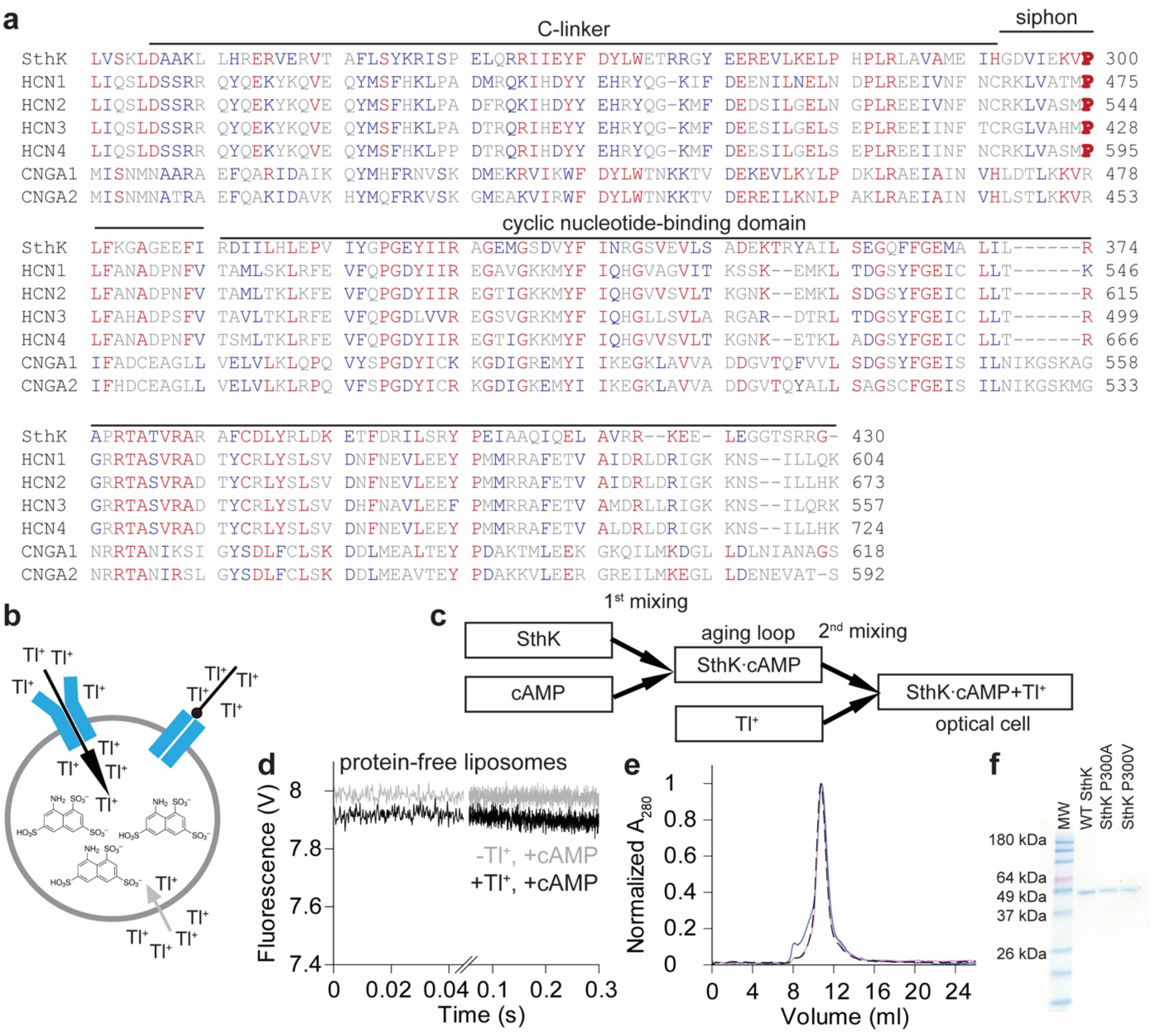

Supplementary Figure 1: Sequence alignment and experimental setup.

a Sequence alignment of the C-linker/CNBD domains of SthK and human HCN and CNG channels for UniProt entries SthK: G0GA88, human HCN1: O60741, human HCN2: Q9UL51, human HCN3: Q9P1Z3, human HCN4: Q9Y3Q4, human CNGA1: P29973, human CNGA2: Q16280. Sequences were aligned using Clustal Omega. Identical residues are colored in red, similar residues in blue, Pro300 (SthK numbering) is highlighted in bold. **b** Schematic representation of SthK reconstituted into ANTS-encapsulating liposomes. Tl^+ serves as quencher for ANTS fluorescence and can enter liposomes quickly through open channels, it cannot enter through closed channels and the leak across the membrane is orders of magnitude slower than through activated channels. Only channels facing the outside with their CNBDs are depicted, since only these channels are sensitive to externally applied cAMP. **c** Mixing scheme of the stopped-flow assay. **d** Fluorescence signal observed for protein-free liposomes in the absence (grey) and presence (black) of Tl^+ after mixing with 200 µM cAMP for 100 ms. **e** Representative gel filtration profiles (Superdex200 10/300) of the three SthK variants used in this study (WT SthK – black, dashed line, SthK P300A – blue, SthK P300V – pink. **f** SDS-PAGE of proteoliposomes containing the three SthK variants (lanes labeled on panel) for the stopped-flow Tl^+ flux assay to verify that channels are present in the liposomes. Only the initial verification experiment was required. MW indicates the molecular weight standard (Benchmark Pre-stained Protein Ladder, Thermo Fisher Scientific).

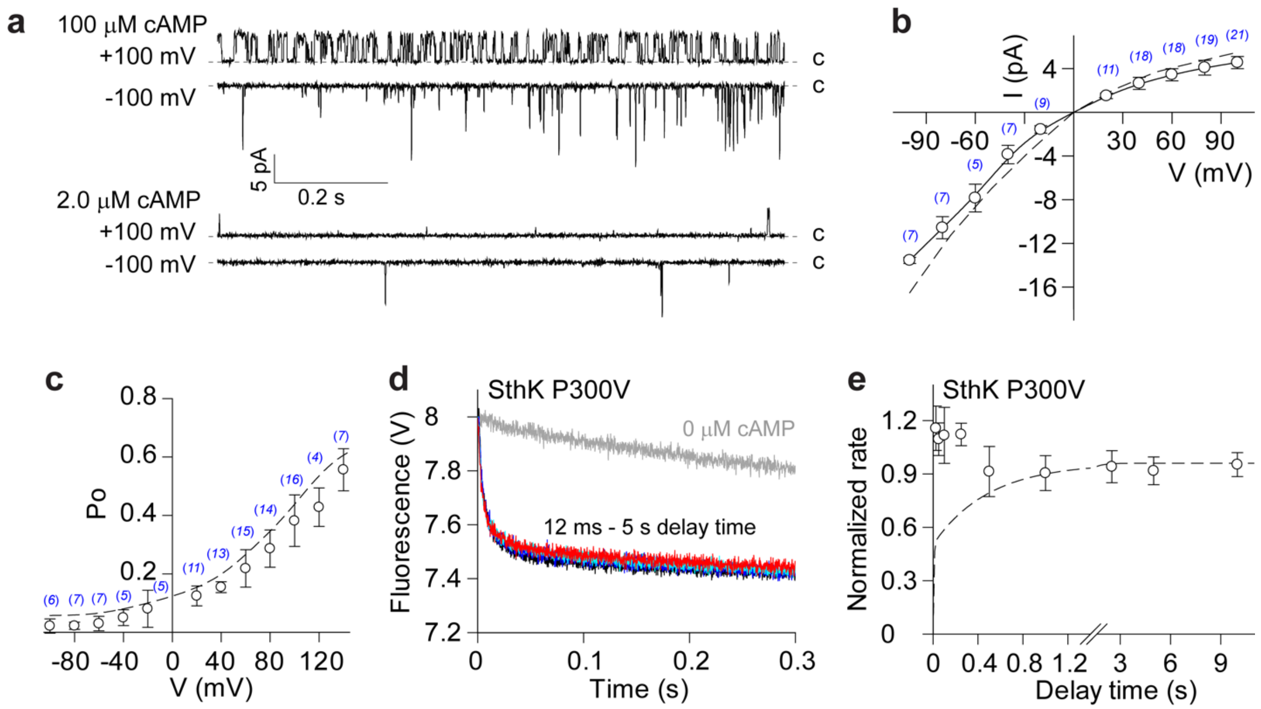

Supplementary Figure 2: Activity of SthK P300A and SthK P300V.

a Representative single-channel recordings for SthK P300A in 10 mM Hepes, 100 mM KCl, pH 7.4 in the presence of 100 μM cAMP (top) and 2 μM cAMP (bottom). Traces are shown for +100 mV and -100 mV. The dashed lines indicate the closed level. **b** I/V relation for SthK P300A in 100 μM cAMP (open circles) and for WT SthK (dashed line, from[1]). **c** Single channel open probability (Po) for SthK P300A in 100 μM cAMP (open circles) and WT SthK (dashed line, from[1]) as function of the applied voltage. In **b** and **c** the number of independent experimental repeats for each data point is indicated above the symbols. **d** Quenching kinetics from the Tl^+ flux assay for SthK P300V in the absence of cAMP (grey) and after incubation with 100 μM cAMP for 12 ms (black), 100 ms (blue), 500 ms (cyan), and 5 s (red). **e** Normalized rates of Tl^+ influx (Equation (2) and (3)) measured for SthK P300V activated by 100 μM cAMP (open circles, n = 3 independent experiments) are plotted as function of the delay time. The dashed line indicates the double exponential fit of the data for WT SthK, taken from Figure 1f. In addition to effects on the activation kinetics of SthK, the absence of slow activation in SthK P300V and SthK P300A (Figure 1e,f) also serves as control that no leakage of cAMP across the vesicular membrane occurs during these experiments (which could lead to additional, slow, activation phases). All symbols represent mean ± S.D. Source data are provided as a Source Data file.

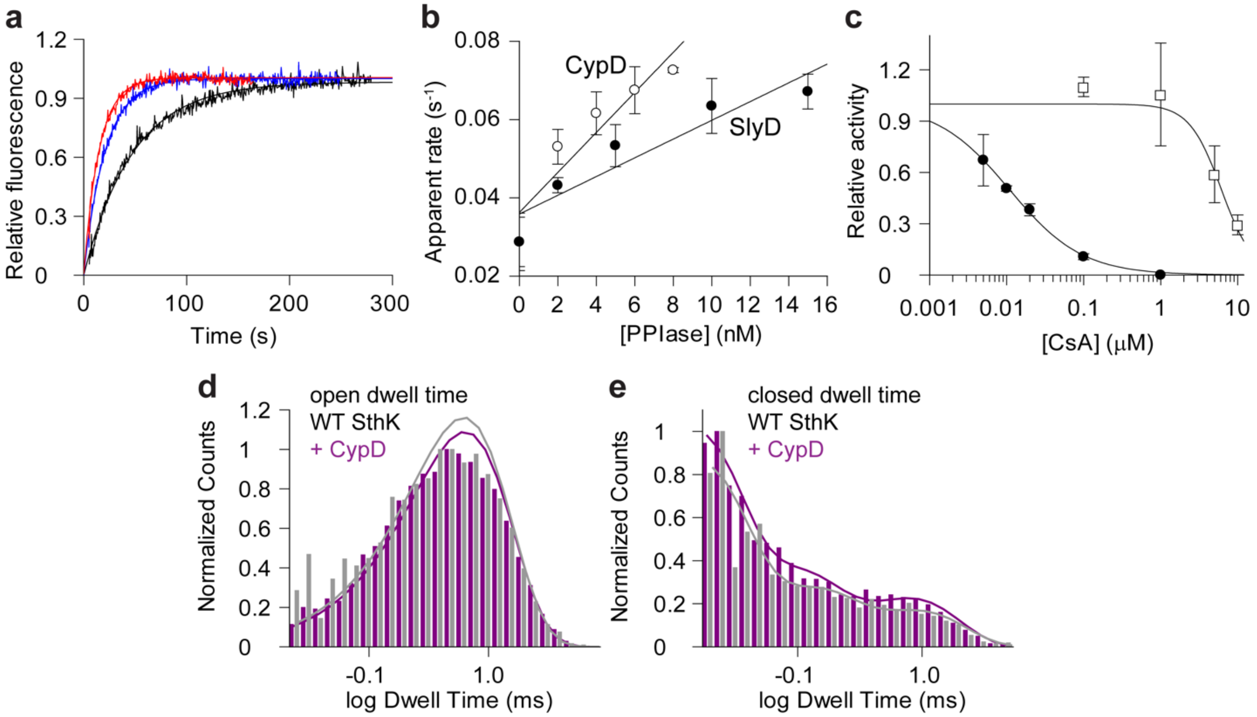

Supplementary Figure 3: Analysis of PPIase activity and the effect on SthK function

a Normalized fluorescence increase in the peptide Abz-ALPF-pNa upon a solvent jump in the absence of CypD (black) and in the presence of 4 nM CypD (blue) and 8 nM CypD (red) in 20 mM Hepes, 100 mM KCl, pH 7.4 at 25 °C. Lines represent fits according to a single exponential function. **b** Apparent rate constants obtained from kinetics as shown in **a** for the substrate Abz-ALPF-pNa in the presence of increasing concentrations of CypD (open symbols, n = 3 independent experiments) or SlyD (filled symbols, n = 3 independent experiments). Lines represent linear fits to determine k_{cat}/K_M according to Equation (6). Numerical values for the catalytic efficiency k_{cat}/K_M are listed in Supplementary Table 1. **c** Inhibition of CypD by CsA obtained with the substrate Abz-ALPF-pNa in 20 mM Hepes, 140 mM KNO_3 in the absence (filled symbols, n = 3 independent experiments) and presence (open symbols, n = 3 independent experiments) of liposomes. Data were fitted according to Equation (5) yielding IC_{50} values of 10.9 ± 0.4 nM in the absence and 6.1 ± 0.7 µM in the presence of liposomes. Symbols in **b** and **c** represent mean ± S.D. **d** Open dwell time distribution of SthK in the presence of 100 µM cAMP, at 100 mV, in the absence (grey) or presence (purple) of 1 µM CypD. Lines represent fits according to a single exponential giving the normalized proportion P and open dwell times τ in the absence of CypD of P = 1 ± 0.03, τ = 4.22 ± 0.05 ms and in the presence of CypD P = 1 ± 0.02, τ = 4.79 ± 0.03 ms. **e** Closed state dwell time distribution for SthK (grey) and SthK in the presence of 1 µM CypD (purple) in the presence of 100 µM cAMP at 100 mV. Lines represent fits with three exponential components. For only SthK results are: P_1 = 0.66 ± 0.07, τ_1 = 0.14 ± 0.13 ms, P_2 = 0.19 ± 0.04, τ_2 = 0.92 ± 0.35 ms, P_3 = 0.14 ± 0.03, τ_3 = 7.5 ± 0.3 ms and for SthK in the presence of CypD P_1 = 0.62 ± 0.02, τ_1 = 0.13 ± 0.06 ms, P_2 = 0.21 ± 0.02, τ_2 = 0.76 ± 0.12 ms, P_3 = 0.16 ± 0.01, τ_3 = 7.4 ± 0.1 ms. Errors are s.e. from the fit. Events longer than 300 ms, which account for only ~1 % of all events, were excluded, as were events shorter than twice the dead time of the data acquisition (0.2 ms). Dwell times were fitted using the exponential, log probability function in Clampfit. The presented dwell times correspond to recordings representative traces from which are shown in Figure 3a. Source data are provided as a Source Data file.

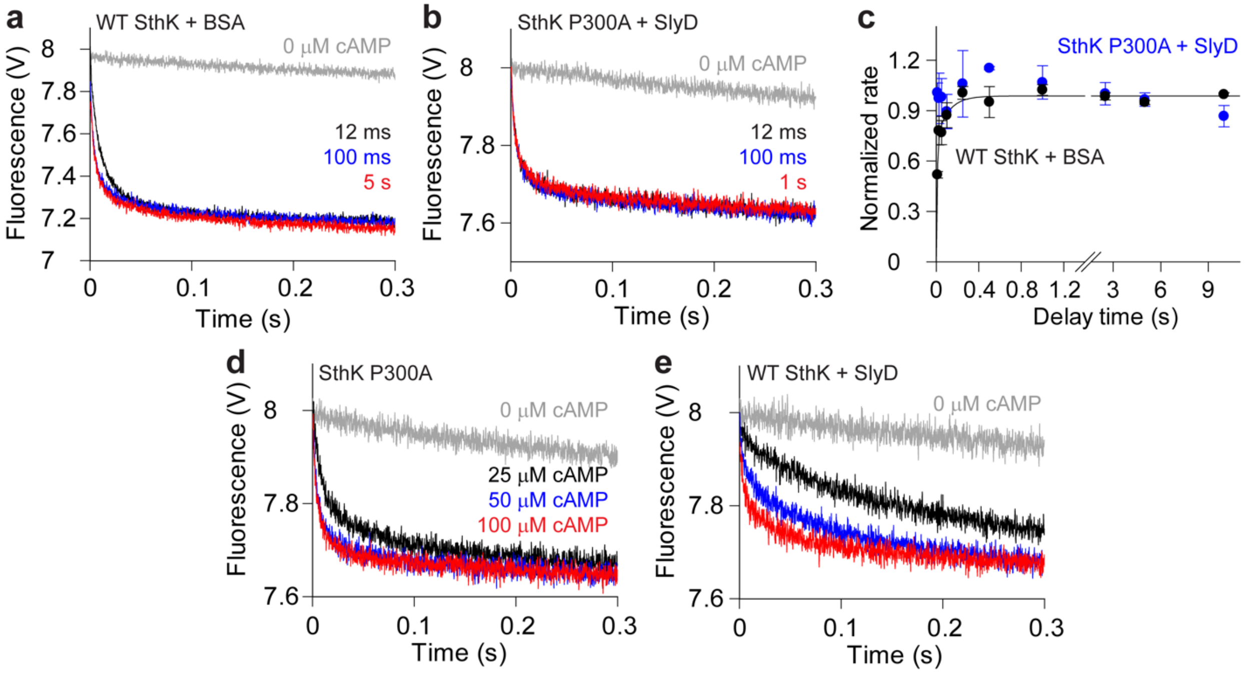

Supplementary Figure 4: Effect of PPIases on SthK function

a Quenching kinetics from the Tl^+ flux assay for WT SthK in the presence of 1 µM purified BSA in the absence of cAMP (grey) and after activation by 100 µM cAMP for 12 ms (black), 100 ms (blue), and 5 s (red). **b** Representative kinetics from the Tl^+ flux assay of SthK P300A in the presence of 1 µM SlyD in the absence of cAMP (grey) and in the presence of 100 µM cAMP after incubation times of 12 ms (black), 100 ms (blue), and 1 s (red). **c** Normalized rate constants of Tl^+ flux (Equation (2) and (3)) after different incubation times with 100 µM cAMP for WT SthK in the presence of 1 µM BSA (black, n = 3 independent experiments) and SthK P300A in the presence of 1 µM SlyD (blue, n = 3 independent experiments). Symbols represent mean ± S.D. The line for WT SthK + 1 µM BSA represents a double exponential fit to the data with amplitudes a and rate constants *k* of $a_1 = 0.7 \pm 0.1$ and $k_1 = 105 \pm 40\ s^{-1}$, $a_2 = 0.3 \pm 0.1$ and $k_2 = 9 \pm 7\ s^{-1}$. While in the presence of BSA the rate of the slow-activating phase is increased, it is still present, reflecting the *cis*/*trans* heterogeneity at Pro300. Furthermore, although BSA was purified by gel filtration before the experiment, there might still be contaminants in the preparation. **d** Quenching kinetics from the Tl^+ flux assay for SthK P300A. Fluorescence was recorded after 2.5 s incubation of the sample with 0 µM cAMP (grey), 25 µM cAMP (black), 50 µM cAMP (blue), and 100 µM cAMP (red). The quenching kinetics show that at 50 µM cAMP SthK P300A is fully activated within the detection range of the assay. Rates obtained from such kinetics according to Equation (2) and (3) are plotted in Figure 3d. **e** Quenching kinetics from the Tl^+ flux assay for WT SthK in the presence of 1 µM SlyD after 2.5 s exposure to 0 µM cAMP (grey), 25 µM cAMP (black), 50 µM cAMP (blue), and 100 µM cAMP (red). With 25 µM cAMP, WT SthK shows intermediate channel activity. With 50 µM cAMP, the activity is almost as pronounced as with 100 µM cAMP (see Figure 3d). Source data are provided as a Source Data file.

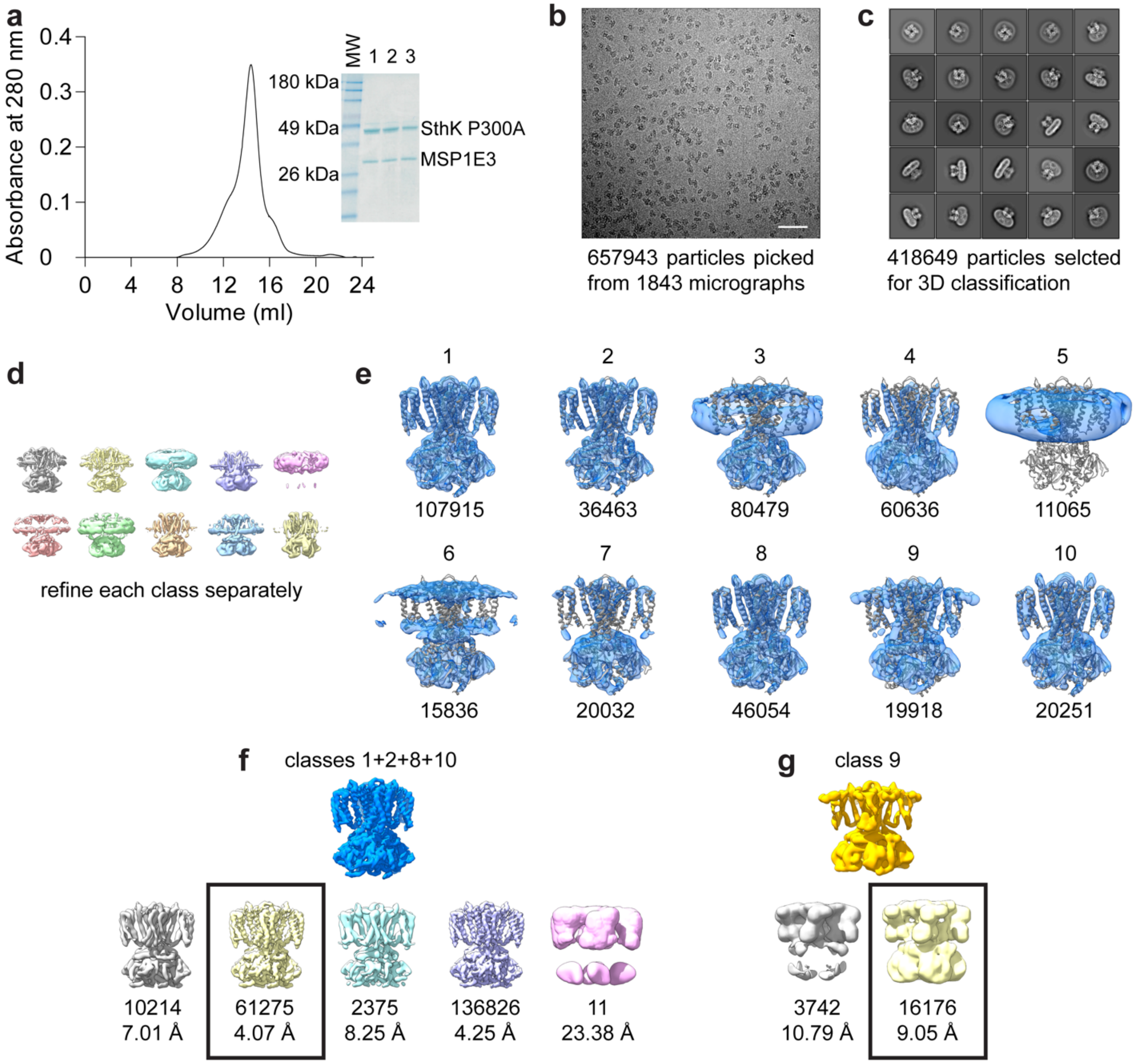

Supplementary Figure 5: cryoEM workflow for SthK P300A

a Gel filtration profile of SthK P300A in nanodiscs and SDS-PAGE analysis of the sample used for the structure determination with cryoEM. MW is the molecular weight standard (Benchmark Prestained, Thermo Scientific), 1-3 are samples taken throughout the main peak (13 – 15 ml elution volume). **b** Representative micrograph of SthK P300A, scale bar is 50 nm. **c** Representative 2D classes (box size 280 Å), **d** 3D classes obtained from unbinned data with a soft mask applied during classification, **e** 10 classes refined and compared to apo SthK (6CJQ[2]), the number of particles is given below each class. **f** Combination of classes that show same features, and results of 3D classification without alignment while a soft mask was applied. Number of particles and estimated resolution are given below each class. **g** Refinement of the putatively active class from the first 3D refinement and the two classes obtained from 3D classification without alignment while a soft mask was applied. Number of particles and estimated resolution are given for both classes. The classes used for final refinements are highlighted in **f** and **g**, also see Supplementary Fig. 6 for final processing details.

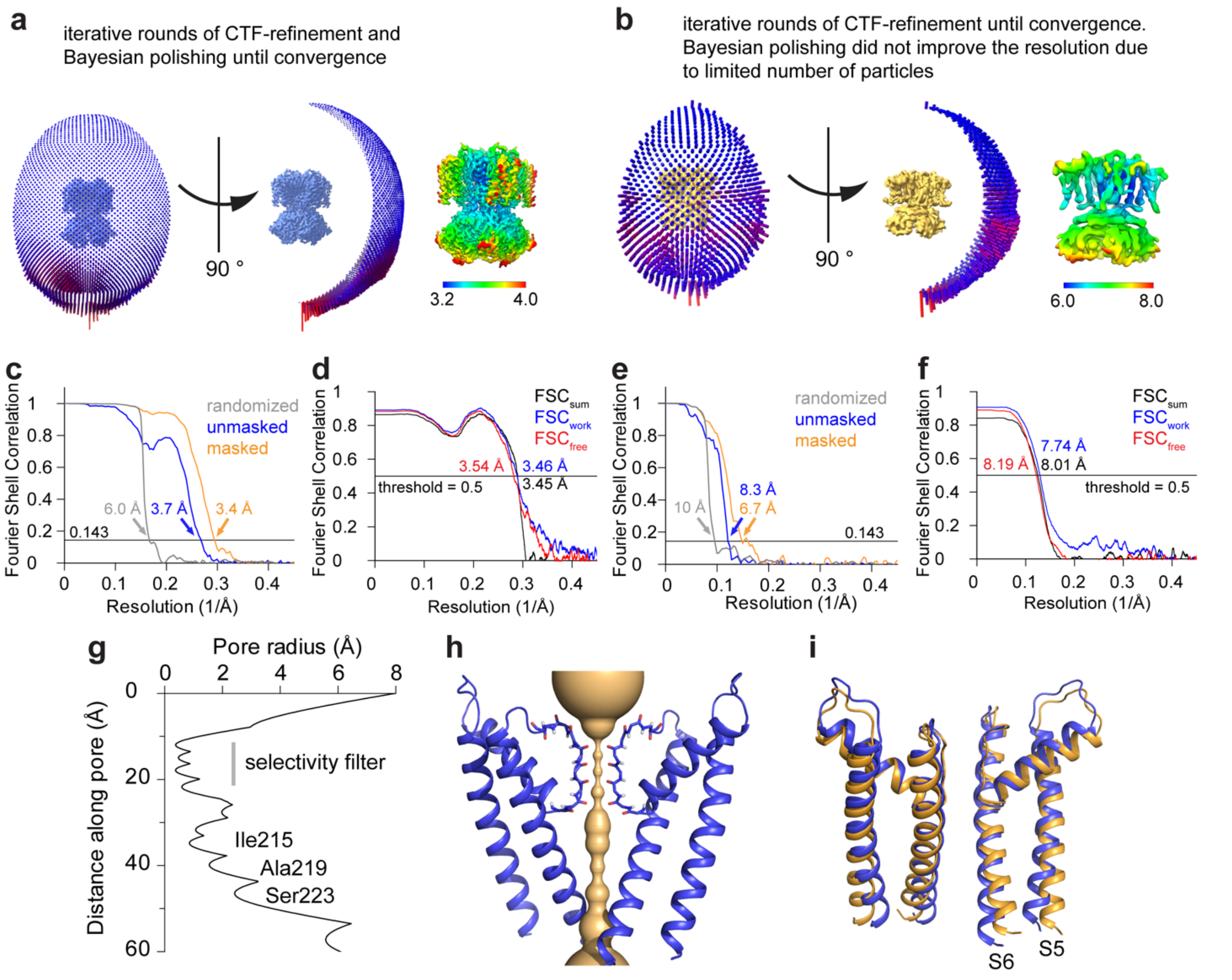

Supplementary Figure 6: Structure and model validation of SthK P300A

Angular distribution plots of the final maps for SthK P300A in the closed state **a** and in the putatively active state **b** shown together with the same maps colored according to the local resolution. **c** FSC curves of the final map for SthK P300A in the closed state. FSC = 0.143 is indicated. **d** Model validation of closed SthK P300A against the final map (FSC_{sum}) and the two half maps (FSC_{work} and FSC_{free}). FSC-threshold of 0.5 is indicated. **e** FSC curves of the final map for the putatively active state of SthK P300A (black line indicates FSC = 0.143). **f** Validation of the model for the putatively active state of SthK P300A against the final map (FSC_{sum}) and the two half maps (FSC_{work} and FSC_{free}). **g** Calculation of the pore radius for SthK P300A in the closed state using the program hole[3], key elements along the pore are indicated. **h** Display of two pore forming units (S5-S6) of SthK P300A (blue) and the pore radius (bronze) as calculated in **g**. **i** S5 and S6 helices of two opposing subunits of SthK P300A in the closed (blue) and putatively open (yellow) state are overlaid, to show widening of the intracellular gate in the putatively open structure.

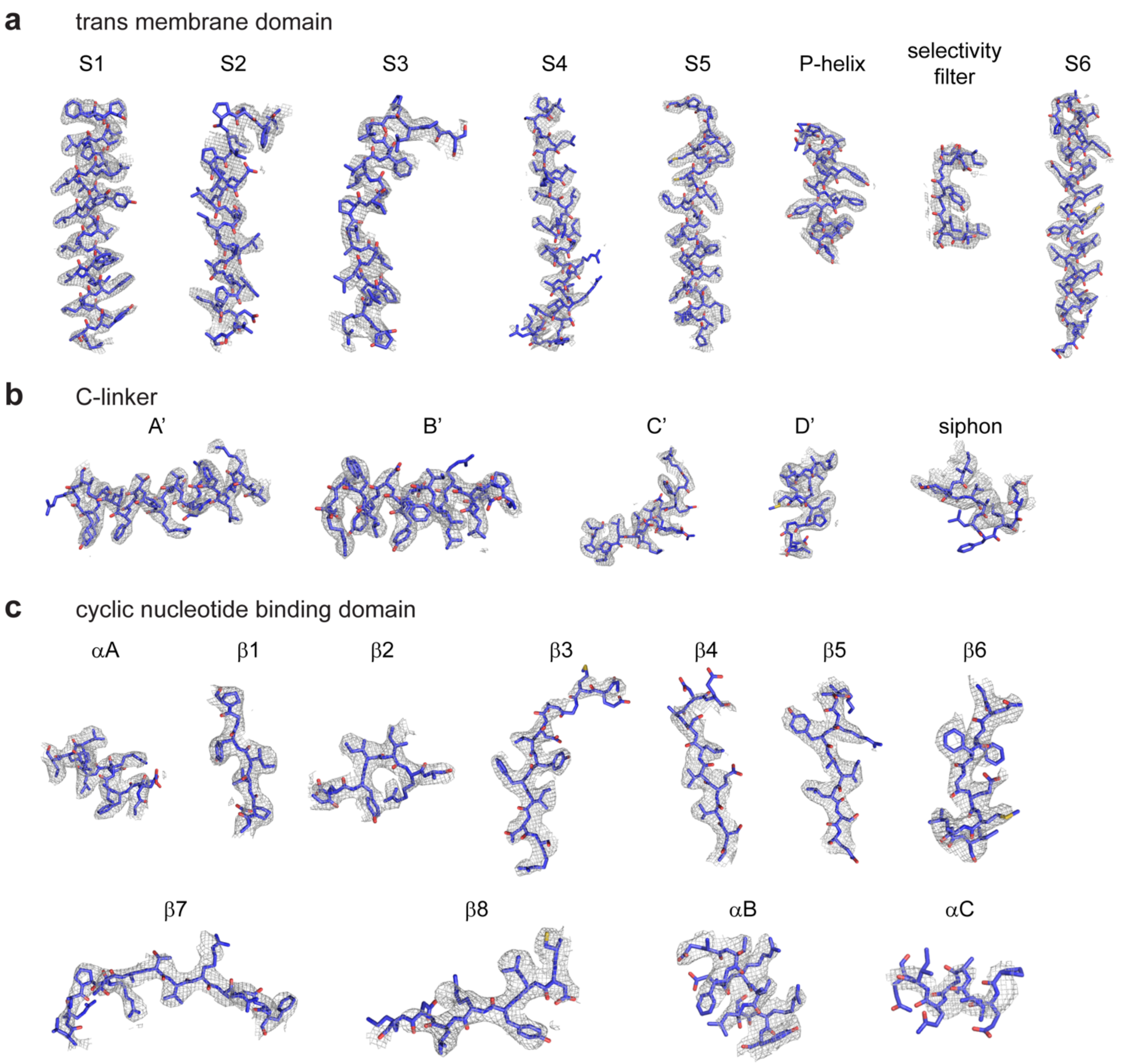

Supplementary Figure 7: Segmented density of SthK P300A in the closed state

The segmented density of SthK P300A bound to cAMP in the closed state is shown in **a** for the trans membrane domain, in **b** for the C-linker, and in **c** for the CNBD. Density is shown in grey as mesh, the model is shown in blue colored by atom.

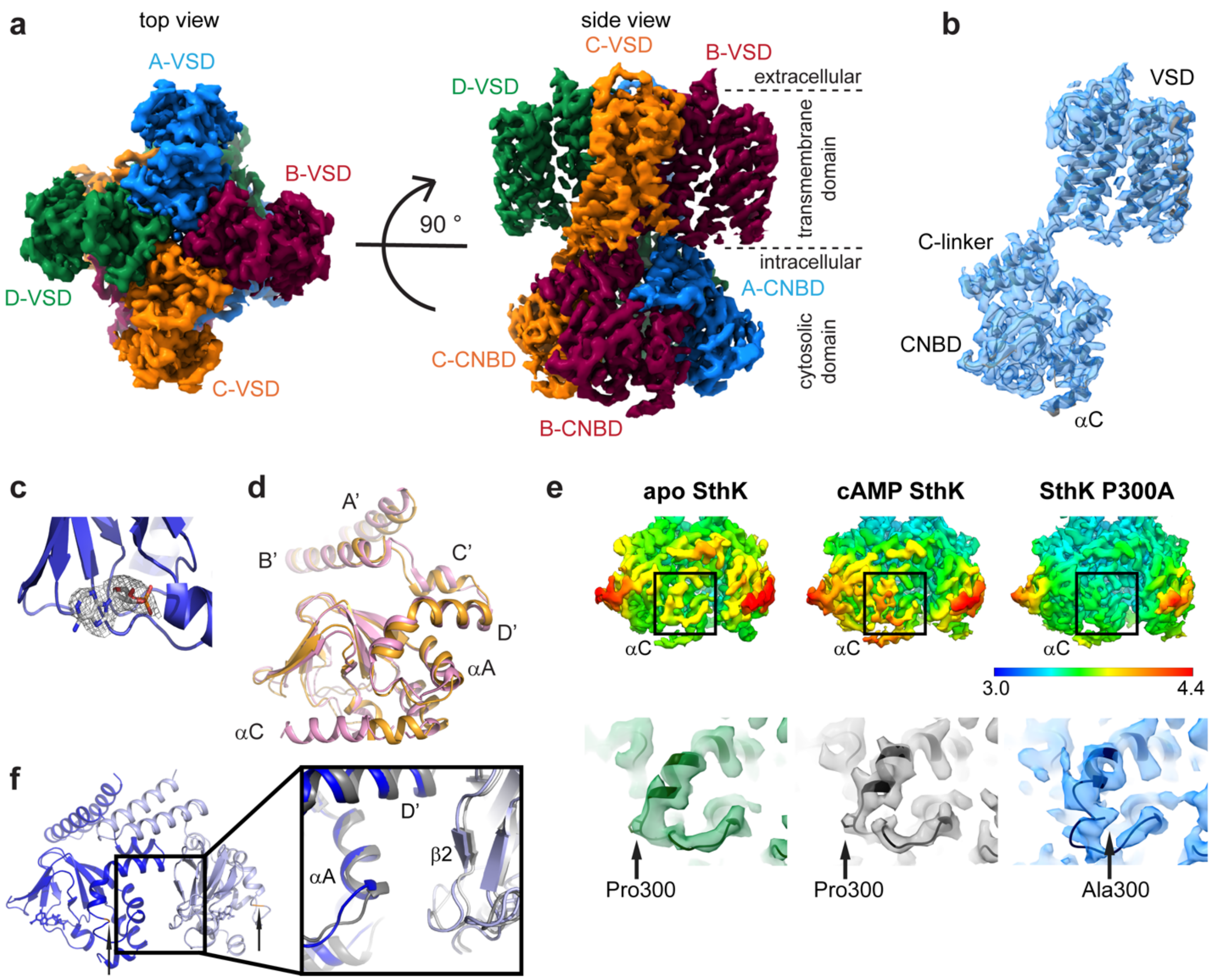

Supplementary Figure 8: Structural features of SthK P300A bound to cAMP

a Density of SthK P300A bound to cAMP in the closed state, colored by subunit. The top view (left panel) shows the tetrameric assembly and the non-swapped VSD domains, the side view (right panel) shows the domain swapping at the level of the cytosolic C-linker/CNBD domains. **b** Density of a single subunit of SthK P300A (blue) bound to cAMP in comparison to the atomic model of WT SthK in the apo state (grey, PDB: 6CJQ[2]). **c** Density map of cAMP (mesh) in the closed state structure of SthK P300A (blue). **d** Overlay of the CNBDs of SthK P300A in the putatively active state (yellow) with the crystal structure of the CNBD bound to cAMP (PDB: 4D7T[4], rose). **e** Local resolution of the C-linker/CNBD as calculated using Relion 3 (top row) for apo WT SthK (EMD-7482[2]), cAMP-bound WT SthK (EMD-7484[2]), and cAMP-bound SthK P300A. All three densities are colored according to the same coloring scheme and the siphon is highlighted by a black square. The bottom row shows the respective zoom into the siphon region. The position of Pro300/Ala300 is indicated. The respective models are shown as cartoon with position 300 in stick representation (apo SthK PDB: 6CJQ[2], cAMP SthK PDB: 6CJU[2]). **f** The C-linker/CNBD of closed state SthK P300A is shown for two subunits (dark blue and light blue) with position 300 in orange (pointed at by arrows) and cAMP in stick representation. The zoom into the CNBD-CNBD interface shows subtle changes in the CNBD-CNBD interface between SthK P300A (blue, all-*trans* mimic) and WT SthK (grey, likely mostly *cis* Pro300, PDB: 6CJU[2]) in the closed state.

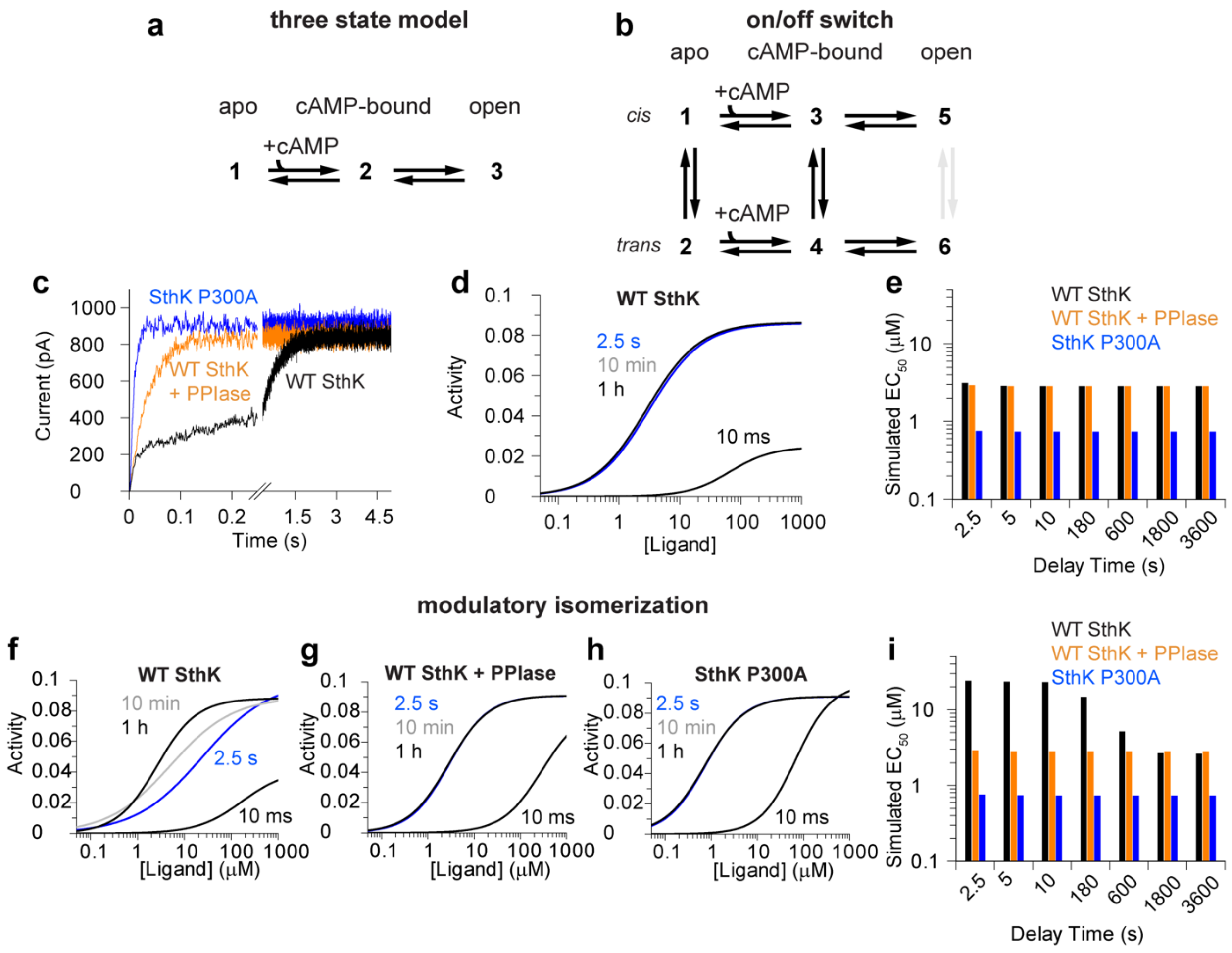

Supplementary Figure 9: Kinetic simulations of the proline switch in SthK

a 3-state model for a ligand gated ion channel. **b** 6-state model that uses prolyl isomerization as on/off switch. Vertical transitions between states 5 and 6 were omitted for simplicity as occupancy of state 5 is exceedingly low. **c** Simulated, macroscopic activation time courses of 10000 channels upon ligand application (100 µM cAMP) for the model shown in **b**. **d** Theoretical dose-response curves for SthK for the model in **b** after different activation times. **e** Calculated EC_{50} values as a function of the delay time for simulations as shown in **d** for the model in **b**. **f**, **g**, **h** Theoretical dose-response curves for WT SthK **f**, SthK + PPIase **g**, and SthK P300A **h** after different times for the model in Figure 5a. **i** EC_{50} values as a function of the delay time (model in Figure 5a). Proteins and delay times are given in the panels. The upper tier in **b** represents the channels with *cis* Pro, the lower tier the ones with *trans* Pro. For simulations of SthK P300A the upper tier was deleted (all-*trans* SthK). To simulate SthK in the presence of PPIase the rates of isomerization (vertical connections) were increased. Channel activity was calculated according to Equation (7) and analyzed with Equation (4). Microscopic rates of the simulations and calculated EC_{50} values are given in Supplementary Tables 3 and 4.

Supplementary Table 1: Summary of PPIase activities

	k_{cat}/K_M (M^{-1} s^{-1}) x 10^6	
	**SlyD**	**CypD**
**20 mM Hepes, 100 mM KCl, pH 7.4**		
Abz-Ala-Leu-Pro-Phe-pNA	2.4 ± 0.4	5.0 ± 0.7
Abz-Ala-Glu-Pro-Phe-pNA	0.006 ± 0.009	3.6 ± 0.2
**20 mM Hepes, 140 mM KNO_3, pH 7.4**		
Abz-Ala-Leu-Pro-Phe-pNA	2.7 ± 0.5	5.1 ± 1.4
Abz-Ala-Glu-Pro-Phe-pNA	0.007 ± 0.01	2.7 ± 0.4

Numerical values of the catalytic efficiency k_{cat}/K_M for the peptide-based isomerization assay are summarized. The different buffer conditions, the different peptides and the different enzymes are indicated. Data correspond to Figure 2f and Supplementary Fig. 3b. All data were acquired at 25 °C.

Supplementary Table 2: cryoEM data processing of SthK P300A and model validation

**Data collection**		
Microscope, camera, magnification	FEI Titan Krios, Gatan GIF/K2, 105000	
Voltage (kV), exposure time total/frame, number of frames per image	300, 10 s/200 ms, 50	
Electron exposure (e^-/Å^2), (e^-/Å^2/frame)	70.98, 1.42	
Defocus range (µm)	-1 to -2.2	
Pixel size (Å)	1.0961	
**Processing**		
Number of images collected, selected	2494, 1843	
Initial number of particles, selected particles after 2D	657943, 418649	
Box size (px)	256	
	SthK P300A closed state	SthK P300A low-populated state
Total number of cleaned particles per class	210683 (91 %)	19918 (9 %)
Particles used for final map	61275	16176
Symmetry imposed	C4	C4
Map resolution (Å) 0.143 FSC threshold	3.42	6.68
Map resolution range (Å)	3.2 – 4	5.8 - 8
Map sharpening B factor (Å^2)	-105	-300
**Model validation**		
MolProbity score, clashscore, poor rotamers (%)	2.38, 12.4, 2.4	1.77, 5.40, 0
Ramachandran plot Favored (%), Allowed (%), Disallowed (%)	91.95, 8.05, 0	92.13, 7.61, 0.26
FSC_{sum}, FSC_{work}, FSC_{free} (threshold = 0.5, masked)	3.45, 3.46, 3.54	8.11, 7.74, 8.19

Summary of the collected cryoEM data, processing details and model validation. Supplementary Table 2 corresponds to Figure 4 and Supplementary Fig. 5, 6, 7, 8.

Supplementary Table 3: Microscopic rate constants used in the simulations

	on/off switch			modulatory isomerization		
α_{ij} (s^{-1})	SthK	SthK + PPIase	SthK P300A	SthK	SthK + PPIase	SthK P300A
α_{12}	1.28854	128.854	n/a	0.00129	129.31	n/a
α_{21}	3.96192	396.192	n/a	0.00395	394.8	n/a
α_{13}	0.023 · p	0.023 · p	n/a	0.023 · p	0.023 · p	n/a
α_{31}	1	1	n/a	1	1	n/a
α_{24}	1.24 · p	1.24 · p	1.24 · p	1.24 · p	1.24 · p	1.24 · p
α_{42}	1	1	1	1	1	1
α_{34}	1.90669	190.67	n/a	n/a	n/a	n/a
α_{43}	0.109614	10.96	n/a	n/a	n/a	n/a
α_{35}	1	1	n/a	100	100	n/a
α_{53}	1000	1000	n/a	1000	1000	n/a
α_{46}	100	100	100	100	100	100
α_{64}	1000	1000	1000	1000	1000	1000
α_{56}	n/a	n/a	n/a	$7.739 \cdot 10^{-4}$	77.39	n/a
α_{65}	n/a	n/a	n/a	$4.387 \cdot 10^{-5}$	4.39	n/a

Microscopic rate constants α_{ij} used in kinetic simulations as shown in Supplementary Fig. 9. Indices indicate the respective transitions from state *i* to *j* according to the models presented in Figure 5a and Supplementary Fig. 9b. n/a indicates that this transition was not considered for the respective simulations. Association reactions of SthK with cAMP are concentration dependent and accordingly the microscopic rates need to be multiplied by the ligand concentration (α_{ij} · p).

Supplementary Table 4: Theoretical EC_{50} values obtained from kinetic simulations

Simulated EC_{50} values (μM)

	on/off switch			modulatory isomerizatoin		
Delay time (s)	WT SthK	WT SthK + PPIase	SthK P300A	WT SthK	WT SthK + PPIase	SthK P300A
2.5	3.1217	2.9069	0.7507	23.8122	2.8709	0.7480
5	2.8526	2.8380	0.7331	23.0994	2.8015	0.7305
10	2.8364	2.8363	0.7325	22.8263	2.7998	0.7299
180	2.8363	2.8363	0.7325	14.5106	2.7998	0.7299
600	2.8363	2.8363	0.7325	5.1268	2.7998	0.7299
1800	2.8363	2.8363	0.7325	2.6491	2.7998	0.7299
3600	2.8363	2.8363	0.7325	2.6125	2.7998	0.7299

Calculated EC_{50} values according to Equation (4) for simulations shown in Figure 5c and Supplementary Fig. 9 are provided. These values were used to generate Supplementary Fig. 9e,i.

Supplementary Table 5: Primers used for cloning and mutagenesis

primer name	5' – 3' nucleotide sequence
SthK_P300A_fwd	ATC GAG AAG GTG GCC CTC TTC AAG GGG
SthK_P300A_rev	CCC CTT GAA GAG GGC CAC CTT CTC GAT
SthK_P300V_fwd	ATC GAG AAG GTG GTC CTC TTC AAG GGG
SthK_P300V_rev	CCC CTT GAA GAG GAC CAC CTT CTC GAT
CypD_NdeI_fwd	GCG TAT CAT ATG TGC AGC AAG GGC
CypD_BamHI_rev	GGC CAG TTG AGC GGT GGA TCA GGG TCG GGA CAT CAC CAT CAC CAT CAC TAG GGA TCC AAT

5' – 3' primer sequences are provided for the mutations P300A and P300V in SthK using Quickchange PCR. The gene for CypD was cloned into pET11a using NdeI and BamHI restriction sites. The His_6-tag used for purification of CypD was introduced with the reverse primer.

Supplementary References

1 Schmidpeter, P. A. M., Gao, X., Uphadyay, V., Rheinberger, J. & Nimigean, C. M. Ligand binding and activation properties of the purified bacterial cyclic nucleotide-gated channel SthK. *J Gen Physiol* **150**, 821-834, doi:10.1085/jgp.201812023 (2018).

2 Rheinberger, J., Gao, X., Schmidpeter, P. A. & Nimigean, C. M. Ligand discrimination and gating in cyclic nucleotide-gated ion channels from apo and partial agonist-bound cryo-EM structures. *Elife* **7**, doi:10.7554/eLife.39775 (2018).

3 Smart, O. S., Goodfellow, J. M. & Wallace, B. A. The pore dimensions of gramicidin A. *Biophys J* **65**, 2455-2460, doi:10.1016/S0006-3495(93)81293-1 (1993).

4 Kesters, D. *et al.* Structure of the SthK Carboxy-Terminal Region Reveals a Gating Mechanism for Cyclic Nucleotide-Modulated Ion Channels. *PloS one* **10** (2015).
