# Supplementary material for: Prolyl isomerization controls activation kinetics of a cyclic nucleotide-gated ion channel
Source: Nat Commun. 2020 Dec 16;11:6401. doi: 10.1038/s41467-020-20104-4 (PMC7744796; doi:10.1038/s41467-020-20104-4)
Supplement: Supplementary file 3 — Reporting Summary [file 41467_2020_20104_MOESM3_ESM.pdf]

nature research

Corresponding author(s): Crina M. Nimigean

Last updated by author(s): 11/09/2020

Reporting Summary

Nature Research wishes to improve the reproducibility of the work that we publish. This form provides structure for consistency and transparency in reporting. For further information on Nature Research policies, see our Editorial Policies and the Editorial Policy Checklist.

Statistics

For all statistical analyses, confirm that the following items are present in the figure legend, table legend, main text, or Methods section.

n/a	Confirmed	
☐	☒	The exact sample size (*n*) for each experimental group/condition, given as a discrete number and unit of measurement
☐	☒	A statement on whether measurements were taken from distinct samples or whether the same sample was measured repeatedly
☒	☐	The statistical test(s) used AND whether they are one- or two-sided<br>*Only common tests should be described solely by name; describe more complex techniques in the Methods section.*
☒	☐	A description of all covariates tested
☒	☐	A description of any assumptions or corrections, such as tests of normality and adjustment for multiple comparisons
☐	☒	A full description of the statistical parameters including central tendency (e.g. means) or other basic estimates (e.g. regression coefficient) AND variation (e.g. standard deviation) or associated estimates of uncertainty (e.g. confidence intervals)
☒	☐	For null hypothesis testing, the test statistic (e.g. *F*, *t*, *r*) with confidence intervals, effect sizes, degrees of freedom and *P* value noted<br>*Give P values as exact values whenever suitable.*
☒	☐	For Bayesian analysis, information on the choice of priors and Markov chain Monte Carlo settings
☒	☐	For hierarchical and complex designs, identification of the appropriate level for tests and full reporting of outcomes
☒	☐	Estimates of effect sizes (e.g. Cohen's *d*, Pearson's *r*), indicating how they were calculated

Our web collection on statistics for biologists contains articles on many of the points above.

Software and code

Policy information about availability of computer code

Data collection	stopped flow: ProDataSX 2.5.1852.0<br>Fluorescence of the peptide based isomerization assay: FelixGX 4.1.0<br>single channel recordings: Clampex 10.7.0.3<br>cryo EM data collection: Leginon 3.3<br>simulations: QUB 2.0.0.34
Data analysis	stopped flow: Matlab 7.12.0.635 (R2011a) and Grafit 5.0.13<br>peptide based isomerization assay: Grafit 5.0.13<br>single channel recordings: ClampFit 10.7.0.3<br>cryo EM data analysis: Relion 3 beta, MotionCor2 CTFFIND-4.1.5 Chimera 1.13.1, ChimeraX 0.91, Phenix 1.17.1-3660, Coot 0.8.9.1, Pymol 1.7.4.0<br>Figure preparation: Adobe Illustrator CS3 13.0.0, and Adobe Illustrator 2020

For manuscripts utilizing custom algorithms or software that are central to the research but not yet described in published literature, software must be made available to editors and reviewers. We strongly encourage code deposition in a community repository (e.g. GitHub). See the Nature Research guidelines for submitting code & software for further information.

Data

Policy information about availability of data

All manuscripts must include a data availability statement. This statement should provide the following information, where applicable:

- Accession codes, unique identifiers, or web links for publicly available datasets
- A list of figures that have associated raw data
- A description of any restrictions on data availability

The maps for SthK P300A in the closed-state and the putatively open state have been deposited in the Electron Microscopy Data Bank (EMDB) under accession codes 21453 (https://www.emdataresource.org/EMD-21453) and 21454 (https://www.emdataresource.org/EMD-21454), respectively. Atomic coordinates for closed state SthK P300A and putatively open SthK P300A have been deposited in the Protein Data Bank (PDB) with accession codes 6VXZ (https://www.rcsb.org/structure/6VXZ) and 6VY0 (https://www.rcsb.org/structure/6VY0), respectively. Structures produced for this study were compared to previously published structures of apo WT SthK (PDB: 6CJQ, https://www.rcsb.org/structure/6CJQ), cAMP-bound WT SthK (PDB: 6CJU, https://www.rcsb.org/structure/6CJU) as well as the cryoEM density for cAMP-bound WT SthK EMD-7484 (https://www.emdataresource.org/EMD-7484). Data were also compared to the isolated C-linker/CNBD of SthK bound to cAMP (PDB: 4D7T, https://www.rcsb.org/structure/4D7T). For all functional data presented in this study (Figure 1, 2, 3, Supplementary Fig. 2, 3, 4) the raw data underlying reported averages are available in the Source data file. The recorded fluorescence and the single-channel recording traces can be obtained from the authors upon reasonable request.

Field-specific reporting

Please select the one below that is the best fit for your research. If you are not sure, read the appropriate sections before making your selection.

☒ Life sciences ☐ Behavioural & social sciences ☐ Ecological, evolutionary & environmental sciences

For a reference copy of the document with all sections, see nature.com/documents/nr-reporting-summary-flat.pdf

Life sciences study design

All studies must disclose on these points even when the disclosure is negative.

Sample size	No statistical analysis was performed that requires a minimum sample size to account for biological variability, in order to reach a specific confidence level. Thus, sample size does not apply here. Sample size (n) was obtained from individual protein expression and purification experiments to guarantee reproducibility.
Data exclusions	stopped flow mixing artifacts were excluded
Replication	each experiment was repeated at least 3 times from independently prepared samples and protein purifications. The exact number of repeats for each experiment is given in the figure legends.
Randomization	Randomization was not performed, since this study does not include cell or animal subjects. Buffer preparation, protein purification, and experiments were always performed under the exact same, tightly controlled conditions (buffer composition, pH, lipid composition, temperature). Randomization is hence not applicable.
Blinding	Neither experimental procedure, nor obtained results were categorized into groups. Furthermore, the presented results are not subjective or prone to be biased by individual investigators. Blinding was thus not necessary.

Reporting for specific materials, systems and methods

We require information from authors about some types of materials, experimental systems and methods used in many studies. Here, indicate whether each material, system or method listed is relevant to your study. If you are not sure if a list item applies to your research, read the appropriate section before selecting a response.

Materials & experimental systems

n/a	Involved in the study	
☒	☐	Antibodies
☒	☐	Eukaryotic cell lines
☒	☐	Palaeontology and archaeology
☒	☐	Animals and other organisms
☒	☐	Human research participants
☒	☐	Clinical data
☒	☐	Dual use research of concern

Methods

n/a	Involved in the study	
☒	☐	ChIP-seq
☒	☐	Flow cytometry
☒	☐	MRI-based neuroimaging
